# Dataset for effect comparison of irrigation by wastewater and ground water on amount of heavy metals in soil and vegetables: Accumulation, transfer factor and health risk assessment

**DOI:** 10.1016/j.dib.2018.04.108

**Published:** 2018-05-02

**Authors:** Elhameh Cheshmazar, Hossein Arfaeinia, Kamaladdin Karimyan, Hooshmand Sharafi, Seyed Enayat Hashemi

**Affiliations:** a Department of Nutrition, School of Public Health, Iran University of Medical Sciences, Tehran, Iran; b Department of Environmental Health Engineering, School of Public Health, Iran University of Medical Sciences, Tehran, Iran; c Environmental Health Research Center, Kurdistan University of Medical Sciences, Sanandaj, Iran; d Department of Environmental Health Engineering, School of Public Health, Tehran University of Medical Sciences, Tehran, Iran; e Students Research Committee, Kermanshah University of Medical Sciences, Kermanshah, Iran; f Department of Environmental Health Engineering, School of Public Health, Bushehr University of Medical Sciences, Bushehr, Iran

**Keywords:** Heavy metals, Vegetables, Health risk, Bushehr

## Abstract

The irrigation source plays an important role in the amount of contaminates in soil and cultivated agricultural products. In this study, the concentration and human health risks of heavy metals (Zn, Mn, Cu, Cr, Cd and Pb) were evaluated in soil, water and vegetables collected from two sites (FGW: Farms irrigated with ground water, FWW: Farms irrigated with wastewater) of Bushehr, Iran. The trend of heavy metals concentration in vegetables from either site was in the following order: Mn ˃ Zn ˃ Cu ˃ Pb ˃ Cr ˃ Cd. Except Cd and Pb, the concentration of heavy metals was in the range of European Union (2006) permissible limit. THQ (Target hazard quotient) values were the highest in Mn followed by the Pb, Cd, Cu, Zn and Cr. Mn, Pb and Cd exceeded safe limit of THQ in several cases, while THQ of other heavy metals was in the range of safe limit. Based on the results, it can be concluded that heavy metals contamination in vegetables grown in Bushehr, especially in FWW site, may pose a great health risks to the local inhabitant through consumption of vegetables. Therefore, it is recommended that the discharge of industrial and municipal wastewater into water resources witch used for vegetable irrigation should be the first step in controlling the level of heavy metals in vegetables.

**Specifications table**

**Table:** 

Subject area	Environmental sciences
More specific subject area	Agricultural sciences
Type of data	Tables and figures
How data was acquired	In this descriptive and analytical study, two cultivation areas were selected on the basis of ground water irrigation (FGW) and wastewater irrigation (FWW). Then, the concentration and human health risks of heavy metals (Zn, Mn, Cu, Cr, Cd and Pb) were evaluated in soil, water and vegetables collected from two selected sites.
Data format	Raw, analyzed
Experimental factors	A quality control was carried out using reagent blanks (HNO_3_/H_2_SO_4_/HClO_4_ ratio 5:1:1), and NIST Standard reference Material 1570A for vegetables, SRM 2709 for soil and RM 1643E for water for heavy metals and delicately analysis of samples via ICP–AES.
Experimental features	The sampling method, samples transfer to the laboratory, samples preparation and analysis of them were performed according to standard method.
Data source location	Bushehr city, Iran
Data accessibility	Data are included in this article

**Value of the data**

• Vegetables are one of the main components of the food basket of people in the world, including Iranians. Therefore, considering their high consumption by humans, their quality control is very important. The data of this study are in line with the above goal
• So far, a similar study has not been done in the study area (Bushehr city). Therefore, the data from this study can provide a basis for future similar studies.
• The data of present study showed that heavy metals contamination in vegetables grown in Bushehr, especially in farms irrigated by wastewater (FWW) site, may pose a great health risks to the local inhabitant through consumption of vegetables.
• The obtained data of this study showed that agricultural lands irrigated with wastewater contained higher levels of heavy metals in their soil compared to those irrigated with ground water (FGW).

## 1 Data

### 1.1 Concentration of heavy metals in the soil and water

Concentrations of heavy metals recorded in water that used for irrigation and also soils from FGW and FWW are given in Table 1. The concentrations of Zn, Mn, Cu, Cr, Cd, and Pb in irrigation water quantified in this study were significantly higher in FWW than in FGW (P < 0.05). The Zn concentration found to be much higher than other heavy metals with the mean values of 121.73 and 78.53 µg L^−1^ in FWW and FGW, respectively, followed by Cr, Pb, Mn, Cu and Cd with concentrations of 53.25 (23.84), 27.29 (12.83), 23.32 (8.24), 17.66 (9.27) and 7.63 (4.31) µg L^−1^ in FWW (and FGW), respectively.

**Table 1:** Mean concentration of heavy metals in soil (mg kg^−1^) and water (µg L^−1^) used for irrigation in FGW (farms irrigated with ground water) and FWW (Farms irrigated with wastewater).

Heavy metals	Soil of FWW site	Soil of FGW site	EU standard^a^	Irrigation water of FWW	Irrigation water of FGW	FAO standard^b^
Zn	68.75	47.71	300	121.73	78.53	200
Mn	277.62	196.12	2000	23.32	8.24	20
Cu	32.68	18.31	100	17.66	9.27	17
Cr	42.19	17.09	100	53.25	23.84	550
Cd	1.76	0.87	3	7.63	4.31	50
Pb	12.04	5.83	100	27.29	12.84	65

a European Union (2006) [1]

b Ayers (1985) [2]

The mean concentrations of heavy metals in the soil were 68.75 (47.71), 277.62 (196.12), 32.68 (18.31), 42.19 (17.09), 1.76 (0.87) and 12.04 (5.83) mg kg^−1^ for Zn, Mn, Cu, Cr, Cd and Pb in FWW (and FGW), respectively (Table 1). Heavy metals content of soil acquired from FWW site was found to be significantly higher compared to FGW site (P < 0.05).

### 1.2 Concentration of heavy metals in the vegetables

Concentrations of heavy metals in the different vegetables collected from two locations of FWW and FGW in Bushehr are summarized in the Table 2. The overall concentrations of heavy metals in vegetables from both sample sites (FWW and FGW) followed the order Mn > Zn > Cu > Pb > Cr > Cd. According to one way ANOVA, all types of vegetables grown in FWW showed significantly higher content of heavy metals compared to those grown in FGW (P < 0.05).

**Table 2:** Mean concentration of heavy metals (mg kg^−1^) in vegetables grown in FGW (farms irrigated with ground water) and FWW (Farms irrigated with wastewater).

Heavy metals	Lettuce	spinach	Cabbage	Onion	Potato	Tomato	Green pepper
Zn^a^	19.75 ± 1.73	23.03 ± 1.80	2.01 ± 0.48	6.3 ± 0.40	3.23 ± 0.17	4.76 ± 0.21	1.62 ± 0.18
Zn^b^	6.46 ± 0.70	13.43 ± 1.36	0.81 ± 0.06	3.21 ± 0.19	1.81 ± 0.10	3.32 ± 0.16	1.17 ± 0.05
Mn^a^	96.06 ± 4.05	37.98 ± 2.23	28.31 ± 1.47	12.96 ± 0.85	6.34 ± 0.74	19.2 ± 1.14	6.41 ± 0.93
Mn^b^	87.76 ± 3.10	27.77 ± 1.43	18.11 ± 1.29	4.31 ± 0.26	3.41 ± 0.48	11.18 ± 0.93	3.43 ± 0.55
Cu^a^	5.12 ± 0.25	7.31 ± 0.69	4.39 ± 0.25	2.81 ± 0.08	4.06 ± 0.22	5.09 ± 0.29	5.87 ± 0.71
Cu^b^	2.81 ± 0.14	3.16 ± 0.24	2.61 ± 0.23	1.48 ± 0.09	3.11 ± 0.19	2.61 ± 0.26	4.12 ± 0.41
Cr^a^	0.41 ± 0.02	0.54 ± 0.07	0.25 ± 0.05	0.31 ± 0.04	0.61 ± 0.05	0.44 ± 0.05	0.38 ± 0.04
Cr^b^	0.26 ± 0.03	0.22 ± 0.04	0.19 ± 0.03	0.26 ± 0.03	0.42 ± 0.03	0.33 ± 0.05	0.24 ± 0.04
Cd^a^	0.57 ± 0.05	1.77 ± 0.12	0.26 ± 0.04	0.13 ± 0.02	0.36 ± 0.04	0.29 ± 0.05	0.41 ± 0.05
Cd^b^	0.13 ± 0.02	0.51 ± 0.05	0.11 ± 0.02	0.06 ± 0.01	0.16 ± 0.03	0.09 ± 0.01	0.13 ± 0.02
Pb^a^	3.13 ± 0.09	3.61 ± 0.10	2.68 ± 0.08	1.73 ± 0.11	1.29 ± 0.09	1.71 ± 0.13	1.51 ± 0.08
Pb^b^	2.62 ± 0.07	2.98 ± 0.15	2.03 ± 0.12	1.19 ± 0.09	0.94 ± 0.07	1.01 ± 0.09	1.04 ± 0.11

a Heavy metal concentration in vegetables from FWW.

b Heavy metal concentration in vegetables from FGW.

**Fig. 1:**
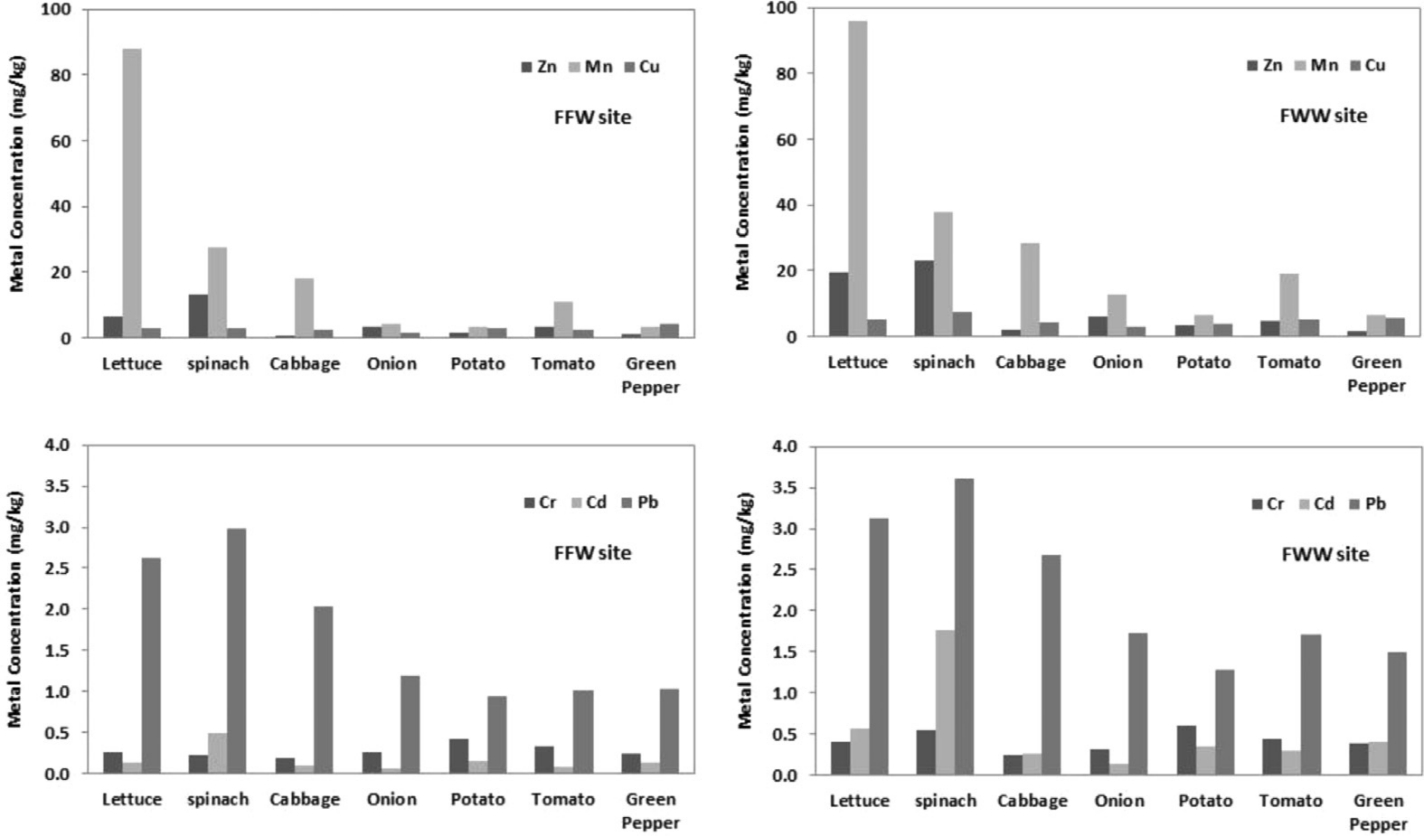
Carcinogenic and essential heavy metals of different vegetables grown in FGW (farms irrigated with ground water) and FWW (Farms irrigated with wastewater).

Concentrations of heavy metals in the edible parts of vegetables collected from two locations of Bushehr are represented in Fig. 1. It can be clearly seen that vegetables grown in FWW site contain higher levels of heavy metals compared to FGW (P < 0.05). The order of carcinogenic heavy metal values in vegetables grown in FGW were Pb > Cr > Cd, except for spinach.

### 1.3 Transfer factor

Transfer factor is defined as metals migration in any form from the soil to the edible part of plants that make them available for consumption. This function is related to several physiochemical factors of soil and plant species that mentioned earlier [3]. In addition, heavy metal transfer factor from soil to the plants is an important agent for human exposure to metals through food chain. Table 3 indicates the transfer factors of heavy metals in vegetables from both sites of the current study. Transfer factors for vegetables grown in FGW site ranged between 0.02–0.28, 0.02–0.45, 0.08–0.23, 0.01–0.02, 0.07–0.57 and 0.20–0.60 for Zn, Mn, Cu, Cr, Cd and Pb, respectively. In this site, the highest transfer factor belonged to Pb in spinach (0.60), followed by Cd in spinach (0.57), Mn in lettuce (0.45), Zn in spinach (0.28) and Cu in green pepper (0.23). The transfer factors for vegetables grown in FWW location ranged 0.02–0.28, 0.02–0.36, 0.09–0.23, 0.01, 0.08–1.04 and 0.11–0.30 for Zn, Mn, Cu, Cr, Cd and Pb, respectively. In FWW site, the greatest value of transfer factor were related to Cd in spinach (1.04), followed by Mn in lettuce (0.36), Zn in spinach (0.33), Pb in spinach (0.30), Zn in lettuce (0.29) and Cu in spinach (0.23). General transfer factor pattern for metals in vegetables of both sites was found to be maximum for Pb, followed by Cd, Cu, Mn, Zn and Cr. The transfer factor for Zn, Cd, Pb, Mn and Cu among various vegetables differed significantly (P < 0.05).

**Table 3:** Transfer factor of heavy metals in vegetables grown in FGW (farms irrigated with ground water) and FWW (Farms irrigated with wastewater).

Heavy metals	Lettuce	Spinach	Cabbage	Onion	Potato	Tomato	Green pepper
Zn^a^	0.29	0.33	0.03	0.09	0.05	0.07	0.02
Zn^b^	0.14	0.28	0.02	0.07	0.04	0.07	0.02
Mn^a^	0.35	0.14	0.10	0.05	0.02	0.07	0.02
Mn^b^	0.45	0.14	0.09	0.02	0.02	0.06	0.02
Cu^a^	0.16	0.22	0.13	0.09	0.12	0.16	0.18
Cu^b^	0.15	0.17	0.14	0.08	0.17	0.14	0.22
Cr^a^	0.01	0.01	0.10	0.01	0.01	0.01	0.01
Cr^b^	0.02	0.01	0.01	0.02	0.02	0.02	0.01
Cd^a^	0.32	1.01	0.15	0.07	0.20	0.16	0.23
Cd^b^	0.15	0.57	0.13	0.07	0.18	0.10	0.15
Pb^a^	0.26	0.30	0.22	0.14	0.11	0.14	0.12
Pb^b^	0.45	0.51	0.35	0.20	0.16	0.17	0.18

a Transfer factor in vegetables from FWW.

b Transfer factor in vegetables from FGW.

### 1.4 Daily intake of metals and target quotients

The Daily intakes of metals (DIM) for adults in the Bushehr through consumption of vegetables are given in the Table 4. Results show that the amounts of metals intake via consumption of vegetables grown in FWW were higher than those from FGW. The trend for DIMs in vegetables grown in FGW was in the order of Mn > Zn > Cu >Pb > Cd > Cr. In FGW site, the highest DIM of Zn (0.00141 mg d^−1^), Cu (0.0069 mg d^−1^), Cd (0.00011 mg d^−1^) and Pb (0.00065 mg d^−1^) were observed in spinach, and the greatest DIM of Mn (0.01917 mg d^−1^) and Cr (0.00009 mg d^−1^) were found in lettuce and potato respectively. Additionally, the general trends for DIMs in vegetables grown in FWW were in the order of Mn > Zn > Cu > Pb > Cd > Cr. In FWW site, the highest DIM of Zn (0.00503 mg d^−1^), Cu (0.00160 mg d^−1^), Cd (0.00039 mg d^−1^) and Pb (0.00079 mg d^−1^) were observed in spinach, and the greatest DIM of Mn (0.02207 mg d^−1^) and Cr (0.00013 mg d^−1^) were found in lettuce and potato respectively.

**Table 4:** Daily intake of metals (DIMs, mg/person/day) in vegetables grown in FGW (farms irrigated with ground water) and FWW (Farms irrigated with wastewater).

Heavy metals	Lettuce	spinach	Cabbage	Onion	Potato	Tomato	Green pepper
Zn^a^	0.00431	0.00503	0.00044	0.00138	0.00071	0.00104	0.00035
Zn^b^	0.00141	0.00293	0.00018	0.00070	0.00040	0.00073	0.00024
Mn^a^	0.02098	0.00829	0.00618	0.00283	0.00138	0.00419	0.00140
Mn^b^	0.01917	0.00606	0.00395	0.00094	0.00074	0.00244	0.00075
Cu^a^	0.00112	0.00160	0.00094	0.00061	0.00089	0.00111	0.00128
Cu^b^	0.00061	0.00069	0.00057	0.00032	0.00068	0.00057	0.00090
Cr^a^	0.00009	0.00012	0.00005	0.00007	0.00013	0.00010	0.00008
Cr^b^	0.00006	0.00005	0.00004	0.00006	0.00009	0.00007	0.00005
Cd^a^	0.00012	0.00039	0.00006	0.00003	0.00008	0.00006	0.00009
Cd^b^	0.00003	0.00011	0.00002	0.00001	0.00003	0.00002	0.00003
Pb^a^	0.00068	0.00079	0.00059	0.00038	0.00028	0.00037	0.00033
Pb^b^	0.00057	0.00065	0.00044	0.00026	0.00021	0.00022	0.00023

a DIMs in vegetables from FWW.

b DIMs in vegetables from FGW.

The target hazard quotient (THQ) for metals through consumption of vegetables grown in FGW and FWW were calculated for adults and values are presented in Table 5. In both sites, the THQ values were found to be the highest for Mn followed by Pb, Cd, Cu, Zn and Cr. In FGW location, the highest THQ of Zn (0.1150), Cd (1.2846), Cu (0.2030) and Pb (1.9141) were observed in spinach, and the highest THQ of Mn (6.8326) and Cr (0.0007) were calculated for lettuce and potato respectively. In the FWW site, spinach exhibited the highest THQ value of Zn (0.1972), Cu (0.4695), Cr (0.0009), Cd (4.5475), Pb (2.3187) and lettuce showed the highest THQ value of Mn (7.8681).

**Table 5:** Target hazard quotient (THQ) for heavy metals in vegetables grown in FGW (farms irrigated with ground water) and FWW (Farms irrigated with wastewater).

Heavy metals	Lettuce	spinach	Cabbage	Onion	Potato	Tomato	Green pepper
Zn^a^	0.1691	0.1972	0.0172	0.0540	0.0277	0.0408	0.0139
Zn^b^	0.0553	0.1150	0.0069	0.0275	0.0155	0.0284	0.0094
Mn^a^	7.4788	2.9570	2.2041	1.0090	0.4936	1.4948	0.4991
Mn^b^	6.8326	2.1620	1.4100	0.3356	0.2655	0.8704	0.2670
Cu^a^	0.3289	0.4695	0.2762	0.1805	0.2608	0.3269	0.3770
Cu^b^	0.1798	0.2030	0.1670	0.0951	0.1998	0.1670	0.2633
Cr^a^	0.0007	0.0009	0.0004	0.0005	0.0010	0.0008	0.0007
Cr^b^	0.0004	0.0004	0.0003	0.0004	0.0007	0.0006	0.0004
Cd^a^	1.4645	4.5475	0.6680	0.3340	0.9249	0.7451	1.0277
Cd^b^	0.3340	1.2846	0.2826	0.1542	0.4111	0.2312	0.3340
Pb^a^	2.0104	2.3187	1.7214	1.1112	0.8286	1.0983	0.9635
Pb^b^	1.6828	1.9141	1.3039	0.7643	0.6038	0.6487	0.6680

a THQ of metals in vegetables from FWW.

b THQ of metals in vegetables from FGW.

## 2 Experimental design, materials and methods

### 2.1 Study area

**Fig. 2:**
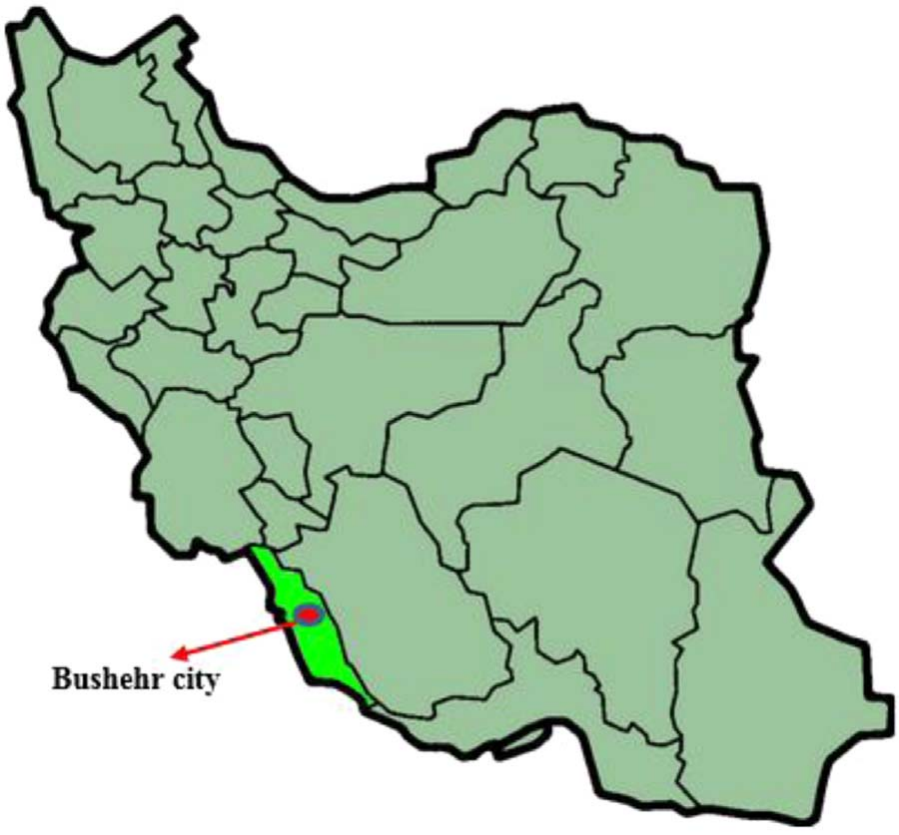
Bushehr city location in Iran map.

Study area, Bushehr city, is located in south of Iran, on the hot Persian Gulf, has a subtropical desert climate, with a mean annual temperature of 24.8 °C, an annual rainfall of 223.2 mm and an average altitude of 28 m [4]. In the county, most farmers have traditionally attended to tomato cultivation. Also, crops are being increasingly variegated with other vegetables and fruits. Various vegetables are now the most important national agricultural products. Two Cultivation areas were selected on the basis of ground water irrigation and wastewater irrigation. Large farmlands irrigated with well water, is located in the south of Bushehr and designated in this study as FGW (farms irrigated with ground water). Farms irrigated with wastewater, designated as FWW, are located near the Khoor River including lands receiving effluents of industries in the vicinity of the river (Fig. 2).

### 2.2 Soil sampling

Soil samples from farm lands were obtained by digging a monolith of 10 × 10 × 10 size using a stainless steel auger. Particles that did not belong to soil such as wooden pieces, stones, organic debris and rocks were removed. All Samples were mixed and dried for 12 h in 100 °C, thereafter grounded to pass 2 mm sieve and kept in poly ethylene bags until analysis. During sampling and storage, it was ensured that samples had not any contact with metal tools for inhibiting cross-contamination [5], [6], [7], [8], [9].

### 2.3 Water sampling

Water samples were collected into pre cleaned bottles that washed earlier with a metal-free soap and eventually rinsed with deionized water. Samples were stored in a 4 °C refrigerator to minimize biodegradation and volatilization prior to analysis [10], [11], [12].

### 2.4 Vegetables sampling

During 2017, a total of 336 samples of harvested fresh vegetables were obtained from different farm lands. Samples were classified into leafy vegetables (lettuce, spinach, cabbage), tuber vegetables (onion, potato), and fruit vegetables (tomato, green pepper). Vegetable samples from each site were collected in three replicates and transported to laboratory in clean polythene bags. The samples were cleaned with distilled water to remove the extraneous materials, then dried in an oven at 100 °C and grinded into powder form to be prepared for digestion [13].

### 2.5 Digestion and analytical procedures

Vegetable samples were digested by a mixture of HNO_3_, H_2_SO_4_ and HClO_4_ at ratio of 5:1:1 at 110 °C until the solution became transparent [14], [15], [16], [17]. The concentrations of Zn, Mn, Cu, Cr, Cd and Pb were quantified using inductively coupled plasma atomic emission spectroscopy (ICP-AES, Arcos model, Germany). Stock standard solutions containing 200 mg L^−1^ for Mn, 50 mg L^−1^ for Pb and Zn, 20 mg L^−1^ for Cu, 5.0 for Cr and 2.0 mg L^−1^ for Cd were applied for preparing working standards. The analysis was operated with radiofrequency power of 1250 W. Delay time was 28 s, and the flow rate of nebulizer, plasma, auxiliary and sample gas were 0.9, 15, 0.2 and 1.6 L min^−1^, respectively. The detection limit (mg kg^−1^) and wavelength (nm) of heavy metals were: 0.006 and 206.2 for Zn; 0.008 and 267.7 for Cr, 0.05 and 221.4 for Pb, 0.004 and 222.8 for Cd, 0.01 and 257.6 for Mn, and, 0.007 and 321.4 for Cu, respectively.

### 2.6 Quality control analysis

All Chemical reagents which used for the sample preparation were of analytical grade and purchased from MERCK Chemicals Germany. A quality control was carried out using reagent blanks (HNO_3_/H_2_SO_4_/HClO_4_ ratio 5:1:1), and NIST Standard reference Material 1570A for vegetables, SRM 2709 for soil and RM 1643E for water for heavy metals and delicately analysis of samples via ICP–AES. The recovery rate for Mn, Cd, Cu, Cr, Zn and Pb were 97.5%, 94.3%, 96.2%, 94.9%, 93.6% and 97.4%, respectively, showing high performance analysis method of heavy metals.

### 2.7 Data analysis

#### 2.7.1 Transfer factor

Transfer factor indicates movement of heavy metals in particular position from the soil to the vegetables [5]. The applied formula was expressed by Cui Y-J et al. [7].

$$\mathrm{TF}=\frac{\mathrm{metal}\;\mathrm{concentration}\;\mathrm{in}\;\mathrm{vegetable}}{\mathrm{metal}\;\mathrm{concentration}\;\mathrm{in}\;\mathrm{soil}}$$

Transfer factor computed in the present study was based on metal concentration of whole edible parts of plant.

#### 2.7.2 Daily Intake of Metals (DIM)

The Daily intake of metals (DIM) was calculated via data achieved using a questionnaire. DIM is related to the heavy metals concentrations in vegetables and the rates of vegetables intake. DIM was calculated in adults by the following equation:

$$\mathrm{DIM}=\frac{C_{\mathrm{metal}}\times C_{\mathrm{factor}}\times W_{\mathrm{food}}}{\mathrm{Bw}}$$

Where C_metal_ represents the heavy metal concentration in vegetables (mg kg^-1^); C_factor_ is conversion factor (0.085); W_food_ is the average of vegetables daily intake; Bw represent the body weight. Our survey on 250 males and females revealed that a local inhabitant consumes averagely 167 g of vegetables per day. The age of participants varied from 31 to 64 year-olds, with an average value of 44 year-olds in male inhabitant; the corresponding data were 27–56 and 39 year-olds in female inhabitant, respectively. In addition, the body weight of participants varied from 55–82 to kg year-olds, with an average value of 71 kg in male inhabitant; the corresponding data were 47–69 and 58 kg in female inhabitant, respectively. In this study the average of body weight in all adult were assumed to be 65 kg.

#### 2.7.3 Target Hazard Quotient (THQ)

Target hazard quotient (THQ) was used to evaluate the local inhabitant health risk of heavy metals from consumption of vegetables. Value less than 1 is considered to be safe for exposed population. The THQ was estimated using method proposed by U.S. EPA Region III risk-based concentration table [12] with the following equation:

$$\mathrm{THQ}=\frac{\mathrm{EF}\;\times \;\mathrm{ED}\;\times \;\mathrm{FI}\times \mathrm{MC}}{\mathrm{RfD}\;\times \;\mathrm{BW}\;\times \;\mathrm{AT}}\;\times {\;10}^{-3}$$

Where THQ represents target hazard quotient; EF is exposure frequency (365 days year^−1^); ED is exposure duration (70 years); FI shows food ingestion (g person^−1^ d^−1^); MC represents metal concentration in food (μg g^−1^, on fresh weight basis); RfD is the oral reference dose (mg kg^−1^ d^−1^) which for Pb, Ni, Cd, Cr, Cu, Mn and Zn is 0.004, 0.02, 0.001, 1.5, 0.04, 0.033 and 0.3 mg kg^−1^ d^−1^, respectively; BW is the average body weight of average adult (65 kg); AT indicates averaging time for noncarcinogens (365 days year^−1^ × number of exposure years, assuming 70 years in this study) [4].
