# Supplementary material for: Dataset for effect comparison of irrigation by wastewater and ground water on amount of heavy metals in soil and vegetables: Accumulation, transfer factor and health risk assessment
Source: Data Brief. 2018 May 2;18:1702–10. doi: 10.1016/j.dib.2018.04.108 (PMC5998744; doi:10.1016/j.dib.2018.04.108)
Supplement: Supplementary file 1 — Supplementary material [file mmc1.docx]

**Compliance with Ethical Standards**

• Authors are aware of, and comply with, best practice in publication ethics specifically with regard to authorship (avoidance of guest authorship), dual submission, and manipulation of figures, competing interests and compliance with policies on research ethics.

• All authors confirm that Boushehr University of Medical Sciences financially supported this study.

• All authors have consented, read and been familiar with this study.

• Authors confirm that research does not involve Human participants or Animals.

• Authors adhere to publication requirements that submitted work is original and has not been published elsewhere in any language.

• All authors present results of the study honestly and without fabrication, falsification or inappropriate data manipulation.

• Authors take collective responsibility for submitted and published work.

• The authors declare no conflict of interest.

Sincerely yours,

Dr.Seyed Enayat Hashemi
